# The gut microbiome as an indicator of habitat disturbance in a Critically Endangered lemur

**DOI:** 10.1186/s12862-021-01945-z

**Published:** 2021-12-16

**Authors:** Nicolette McManus, Sheila M. Holmes, Edward E. Louis, Steig E. Johnson, Andrea L. Baden, Katherine R. Amato

**Affiliations:** 1 Department of Anthropology, Northwestern University, Evanston, IL 60208 USA; 2 Department of Anthropology and Archaeology, University of Calgary, Calgary, AB T2N 1N4 Canada; 3 Department of Wildlife, Fish and Environmental Studies, Swedish University of Agricultural Sciences, 90183 Umeå, Sweden; 4 Grewcock Center for Conservation and Research, Omaha’s Henry Doorly Zoo, Omaha, NE 68107 USA; 5 Department of Anthropology, Hunter College of the City University of New York, New York, NY 10065 USA; 6 Department of Anthropology, The Graduate Center of the City University of New York, New York, NY USA; 7 The New York Consortium in Evolutionary Primatology (NYCEP), New York, USA

**Keywords:** *Varecia*, Madagascar, Diet, Host-microbe, Conservation

## Abstract

**Background:**

Habitat disturbance affects the biology and health of animals globally. Understanding the factors that contribute to the differential responses of animals to habitat disturbance is critical for conservation. The gut microbiota represents a potential pathway through which host responses to habitat disturbance might be mediated. However, a lack of quantitative environmental data in many gut microbiome (GM) studies of wild animals limits our ability to pinpoint mechanisms through which habitat disturbance affects the GM. Here, we examine the impact of anthropogenic habitat disturbance on the diet and GM of the Critically Endangered black-and-white ruffed lemur (*Varecia variegata editorum*). We collected fecal samples and behavioral data from *Varecia* occupying habitats qualitatively categorized as primary forest, moderately disturbed forest, and heavily disturbed forest.

**Results:**

*Varecia* diet and GM composition differed substantially across sites. Dietary richness predicted GM richness across sites, and overall GM composition was strongly correlated to diet composition. Additionally, the consumption of three specific food items positively correlated to the relative abundances of five microbial strains and one microbial genus across sites. However, diet did not explain all of the GM variation in our dataset, and differences in the GM were detected that were not correlated with diet, as measured.

**Conclusions:**

Our data suggest that diet is an important influence on the *Varecia* GM across habitats and thus could be leveraged in novel conservation efforts in the future. However, other factors such as contact with humans should also be accounted for. Overall, we demonstrate that quantitative data describing host habitats must be paired with GM data to better target the specific mechanisms through which environmental change affects the GM.

**Supplementary Information:**

The online version contains supplementary material available at 10.1186/s12862-021-01945-z.

## Background

Increasingly, wild animals are being confronted with rapidly changing environments due to habitat degradation, climate change, and other anthropogenic factors. While these environmental insults have negative effects on the health and survival of a range of taxa [1–5], primates have been particularly impacted, with an estimated sixty percent of the world's primates currently in danger of extinction [6]. Nevertheless, the magnitude of impact varies across species. Even within the order Primates closely related taxa exposed to the same disturbance can exhibit distinct outcomes. For example, in Colombia, the brown spider monkey (*Ateles hybridus*) exhibits a more marked stress response to logging and is at greater risk for extinction compared to the sympatric red howler monkey (*Alouatta seniculus*) [7]. Understanding the factors that contribute to the differential responses of primate species to habitat disturbance is critical for conservation. A wide body of literature addressing this topic suggests that factors such as life history and dietary niche are key in determining these outcomes [8, 9]. However, critical gaps remain in our knowledge of the mechanisms driving these dynamics. As a result, it is often difficult both to predict the impact of specific disturbances on animal health and survival, and to design effective interventions accordingly.

The gut microbiome (GM) represents a novel perspective for understanding host responses to habitat disturbance [10, 11]. Anthropogenic habitat disturbance can alter food availability and diet, disrupt social structure and dispersal patterns, and increase exposure to humans, domestic animals, and associated pathogens [6]. These factors can directly influence host physiology, for instance, by increasing stress [2, 3, 12], reducing food availability [1, 13], and altering infectious disease landscapes [14, 15]. However, they can also affect the GM [16–24], which contributes to host nutrition and metabolism, immune function, and behavior [25–27]. Therefore, the magnitude and direction of GM change may contribute strongly to host outcomes in degraded environments. For example, the GM can provide hosts with key services–including the degradation of dietary fibers and toxins–that increase the nutritional accessibility of food items [28]. Therefore, differences in the GMs of populations occupying distinct habitats could reflect local adaptation, including differences in GM functions that allow consumption of habitat-specific food items. Nevertheless, the primate GM is constrained by host phylogeny and associated physiological adaptations, which may limit the extent to which it can enable marked changes in host diet [29]. Furthermore, the reduced GM diversity associated with reduced dietary diversity for several primate species in degraded habitats suggests a loss of microbial function instead of a change or gain [16, 30, 31]. In these cases, changes in the GM may actually compound the nutritional challenges experienced by these primates. An increased understanding of these dynamics could facilitate the use of the GM as a biomarker for understanding primate responses to habitat disturbance and/or a novel target for the development of interventions [32–34].

Compared to many wild animals, primates are well-studied with regard to host-GM interactions [35]. As a result, we know that some primate species exhibit marked GM differences in response to habitat disturbance. Red colobus monkeys (*Procolobus gordonorum*) in the Udzungwa Mountains of Tanzania and black howler monkeys (*Alouatta pigra*) in Palenque National Park, Mexico exhibit less diverse GMs with higher relative abundances of potential pathogens and lower relative abundances of potentially beneficial taxa when sampled in small forest fragments versus larger, less-disturbed patches of forest [16, 31]. Similarly, the GMs of lowland gorillas (*Gorilla gorilla*) in the Dzanga-Sangha protected areas of Central African Republic can be distinguished based on anthropogenic exposure [17]. In contrast, mantled howler monkeys (*Alouatta palliata*) in Nicaragua and Costa Rica as well as omnivorous ring-tailed lemurs (*Lemur catta*) at the Bezà Mahafaly Special Reserve in southwestern Madagascar show minimal GM differences across a gradient of habitat degradation [36, 37]. While these distinct patterns could be a result of different host ecologies and their interactions with the GM, they may also reflect limitations in study design. Because anthropogenic disturbance is not clearly defined or quantified in most studies, it is often unclear what underlying environmental and/or host factors are being tested. Although most researchers assume that differences in diet across habitat types are important contributors to the reported GM patterns, diet is rarely measured [but see 16]. Furthermore, other factors such as proximity to roads or human settlements and prevalence of logging or hunting are often not considered. More explicit quantification of environmental factors across habitats is necessary to identify generalizable principles describing how the GM interacts with host biology and ecology in anthropogenically-impacted habitats.

To contribute to this goal, here we quantify the relationship between diet and the GM in Critically Endangered black-and-white ruffed lemurs (*Varecia variegata editorum*) occupying three habitats with different types and magnitudes of anthropogenic disturbance. *Varecia* are endemic to Madagascar’s eastern lowland and mid-altitude rain forests [38, 39] and are considered obligate frugivores, consuming 75–99% fruit in pristine forest sites, though their degree of frugivory varies seasonally [40–44]. Given their selective feeding habits, *Varecia* are especially susceptible to anthropogenic pressure that often results in reduced fruit availability [42, 45, 46]. Although they are known to broaden their diets in disturbed forests to include more introduced plant species as well as a larger proportion of leaves [47–50], these diets may alter intake of essential nutritional resources [42, 51], and *Varecia* are usually among the first species to disappear from disturbed habitats [45, 47, 52]. Knowledge of how the GM responds to these changes in *Varecia* dietary landscapes is important for understanding potential mechanisms of dietary plasticity and, ultimately, predicting outcomes in disturbed habitats. Recent work suggests that both diet and other unmeasured aspects of habitat disturbance may play a role in shaping the *Varecia* GM [30], but without paired fecal and behavioral data from the same individuals, interpretation of these patterns remains limited.

**Fig. 1:**
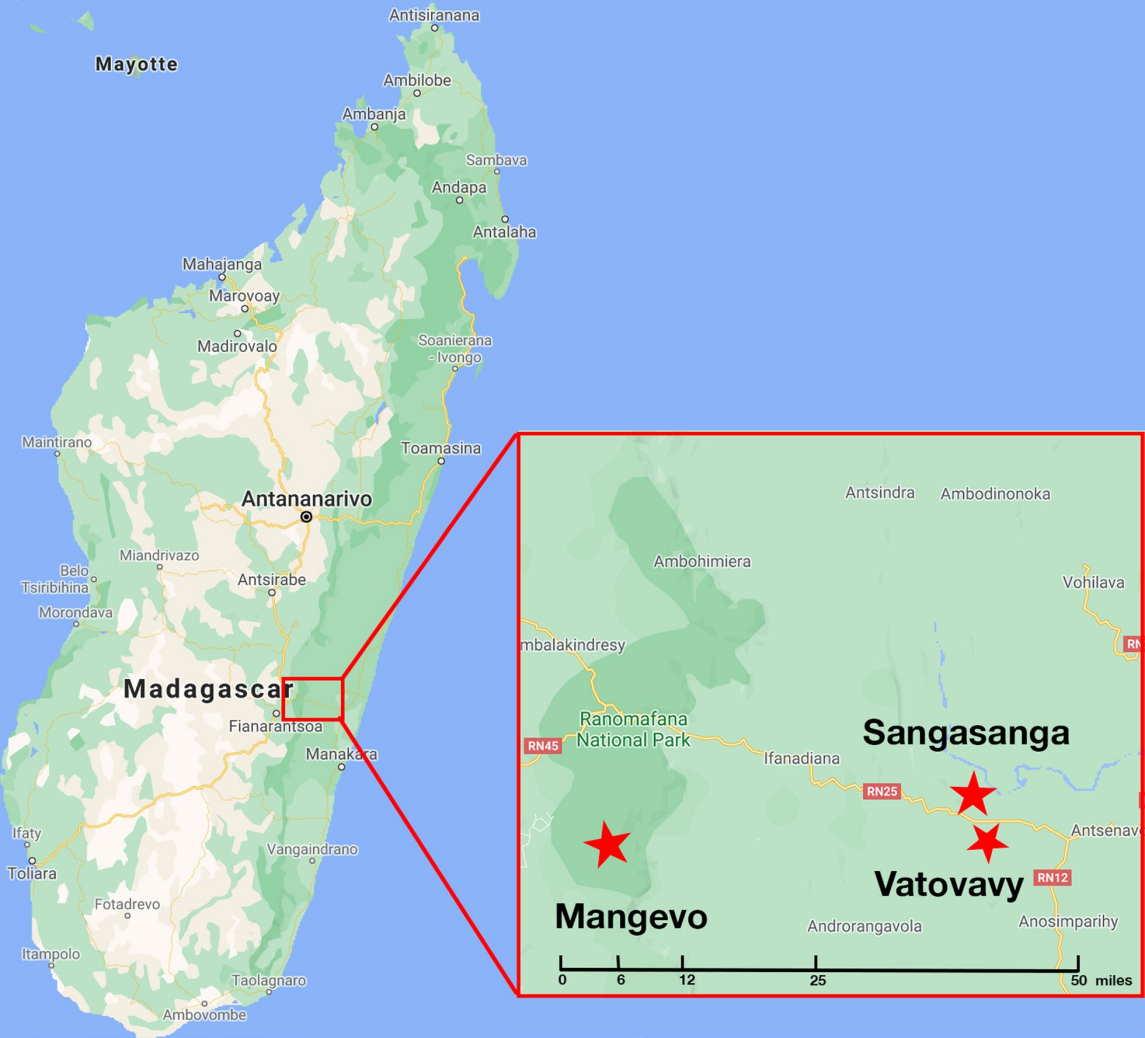
Map of sites. (note to editor: this is our own image)

To determine the extent to which variation in diet across habitats shapes the GM, we compared focal-individual behavioral data paired with 16S rRNA gene amplicon sequencing data of fecal samples from *Varecia* in three geographically proximate populations: a primary forest (Mangevo, Ranomafana National Park), a moderately disturbed forest (Vatovavy), and a heavily disturbed forest (Sangasanga) (Fig. 1, Table 1). We hypothesized that habitat degradation would affect both the *Varecia* diet and GM and that variation in diet would explain a large proportion of the variation in the GM across sites. Specifically, we predicted that in both degraded habitats, *Varecia* would consume similar, less diverse diets with reduced proportions of fruit and exhibit similar, less diverse GMs. We also predicted that across habitats, individual dietary richness would predict individual microbiome richness and that the consumption of specific food items would be associated with variation in the relative abundances of specific microbial taxa (e.g., increased leaf consumption predicts increased relative abundance of fiber-degraders such as *Roseburia*).

**Table 1:** Characteristics of each of the three field sites at which *Varecia variegata* was studied

	Mangevo	Vatovavy	Sangasanga
Disturbance level (categorical)	Undisturbed	Moderately disturbed	Heavily disturbed
Stem density (trees > 10 cm DBH, stems/ha)	699.3	358.92^b^	344.52^b^
Mean DBH (trees > 10 cm DBH, cm)	23.81	23.15^b^	24.76^b^
Mean height (trees > 10 cm DBH, m)	14.09	11.49^b^	10.87^b^
Mean canopy openness (%)	21.4	46.00^b^	59.70^b^
Trees cut down	5.4 trees/ha^a^	49% of transect area had at least some trees cut	55% of transect area had at least some trees cut
Signs of fire	0^a^	19% of transect area had at least some burning	0% of transect area burned
Distance to nearest human settlement	> 3 km	< 2 km	< 1 km
Distance to nearest forest patch	na	5.89 km	< 0.1 km

Mangevo data were collected in 2019 (A. Baden and A. Mancini, unpublished) and Vatovavy and Sangasanga data in 2018 (E. Louis, unpublished), unless otherwise indicated

^a^2004, P. Wright and S. Johnson, unpublished

^b^2014, E. Louis and D. Rafidimanana, unpublished

## Results

### Behavioral data

**Fig. 2:**
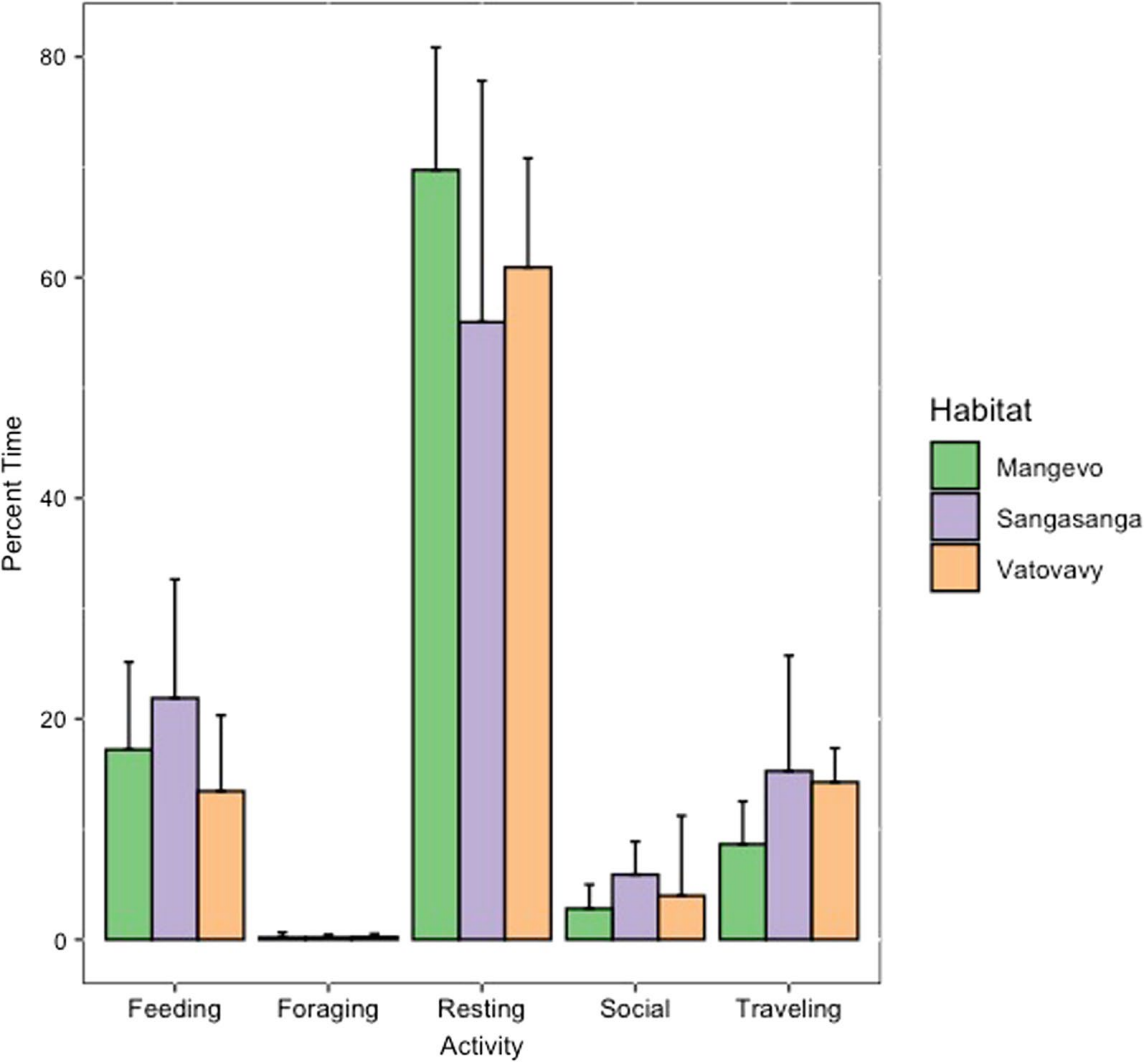
Average percent time (± SD) spent in each recorded behavior at each site during the study period

**Fig. 3:**
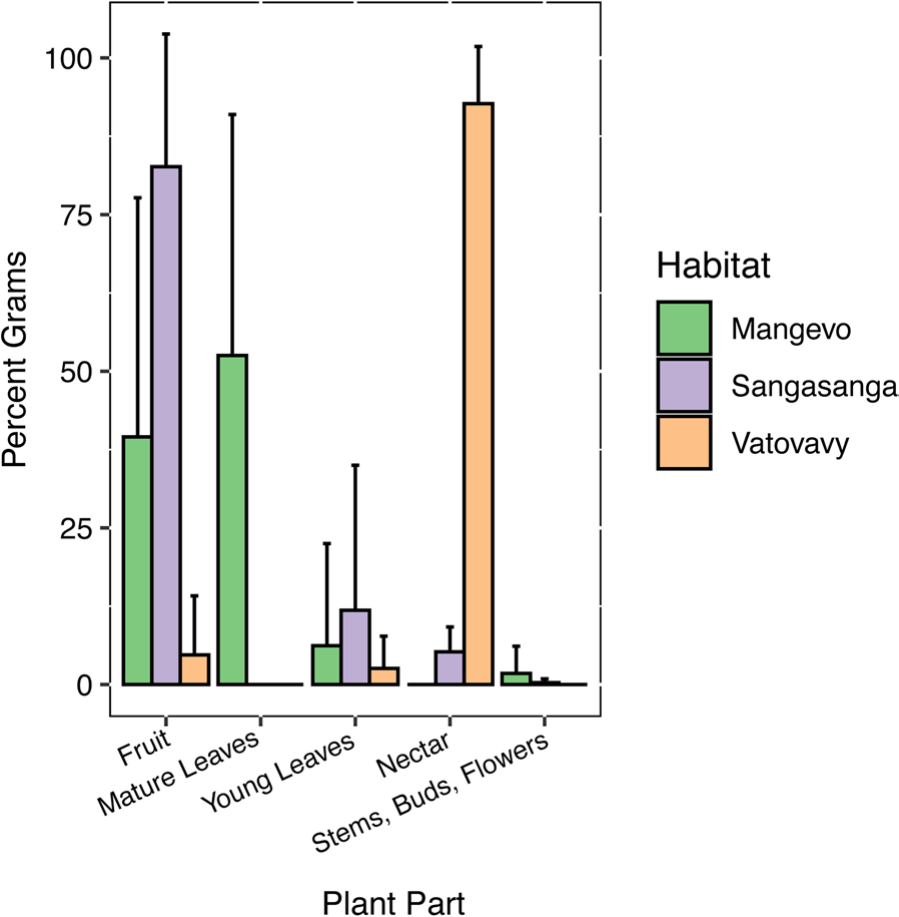
Average percent grams (± SD) consumed for each major plant part in the *Varecia* diet at each site during the study period

**Fig. 4:**
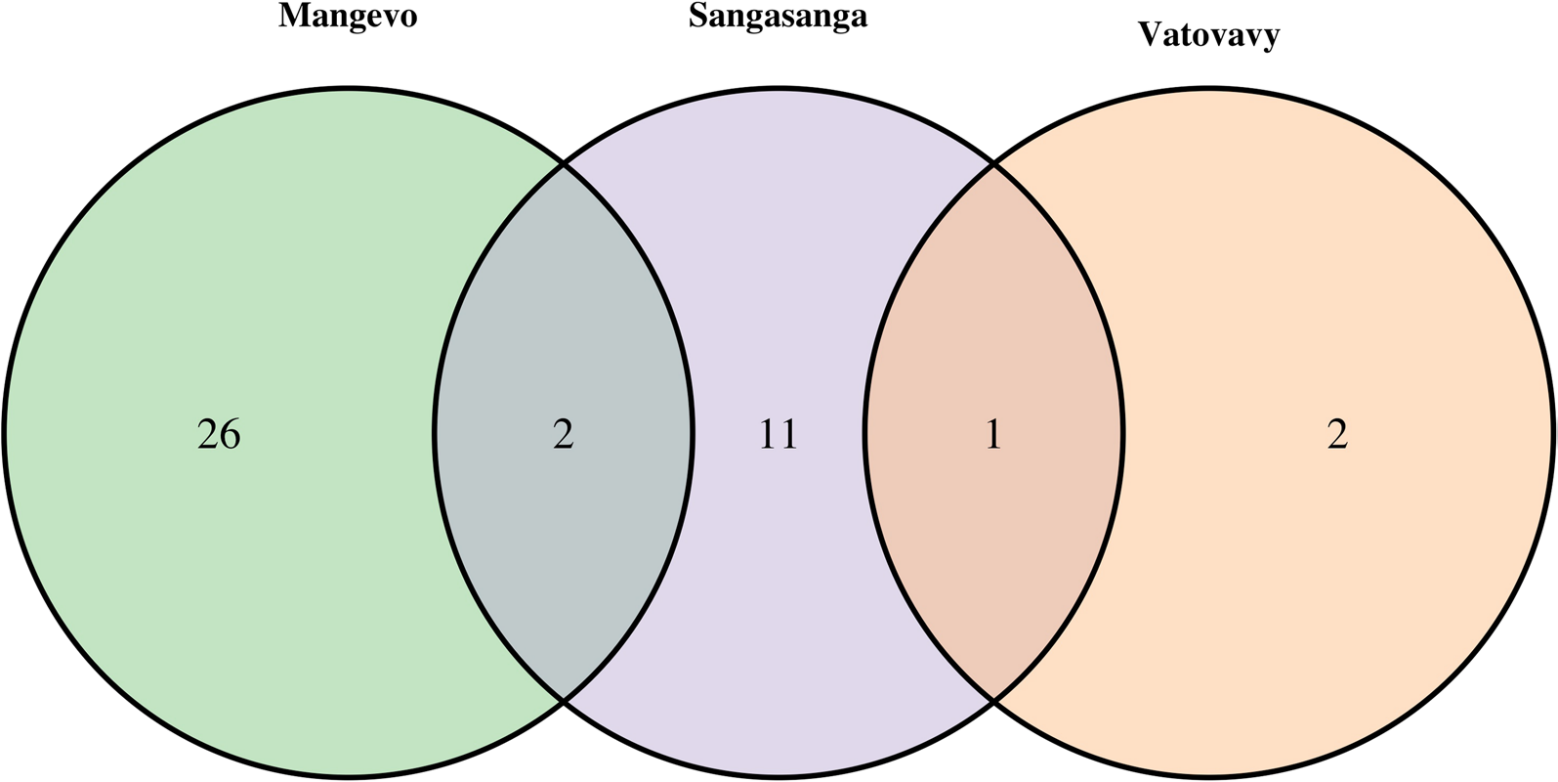
Venn diagram depicting differences in the foods (plant species and part) consumed at each site during the study period

*Varecia* activity budgets were similar for individuals across sites (Fig. 2). There were no significant differences in percent time spent resting, feeding, foraging, traveling, or engaging in social behavior (Additional file 2: Table S1). However, the proportion of food items making up the diet of individuals at each site varied (Additional file 2: Table S2). Mature leaves made up a higher proportion of the diet in Mangevo compared to the other sites (χ^2^ = 12.7, df = 2, p = 0.004), while nectar was higher in Vatovavy (χ^2^ = 18.1, df = 2, p = 0.00004; Fig. 3). The least amount of fruit was consumed in Vatovavy (χ^2^ = 7.4, df = 2, p = 0.01; Fig. 3). Overall, dietary richness was greatest in the undisturbed site, and there was little overlap in the plant species consumed across sites (Fig. 4, Table 2). The only two food items consumed at more than one site were *Canarium madagascariensis* fruit in both Mangevo and Sangasanga, and *Ravenala madagascariensis* nectar in both Vatovavy and Sangasanga. At the individual level, dietary richness differed significantly across sites (F_2,17_ = 4.1, p = 0.04). However, in contrast to patterns at the group level, individuals in Sangasanga consumed more food items on average (5.5 ± 2.0 food items) compared to individuals in Mangevo (4.7 ± 3.4 food items), while individuals in Vatovavy consumed fewer food items on average (1.5 ± 0.6 food items).

**Table 2:** Food items consumed at each site, including the size of the plant part as well as the the observed size of each bite and consumption rate

Site	Family	Plant genus	Plant sp.	Common name	Plant part	Exotic	Plant part size (g)	Bite size (g)	Consumption rate (bites/min)
Mangevo	Rubiaceae	Adena	Microcephala	Voakiringy	Fruit	–	0.6	0.6*	1.42
Mangevo	Loranthaceae	Bakerella	Clavata	Tongolahy	Mature leaves	–	0.66	0.33	3.85
Mangevo	Burseraceae	Canarium	Madagascariensis	Ramy	Fruit	–	0.5	0.5*	0.55
Mangevo	Apocynaceae	Carissa	Edulis	Fantsy	Fruit	–	52.5	17.5	0.61
Mangevo	Lauraceae	Cryptocarya	Acuminata	Tavolomalady	Mature leaves	–	2.08	0.52*	2.41
Mangevo	Lauraceae	Cryptocarya	Ovalifolia	Tavolomanitra	Mature leaves	–	2.08	0.52	3.31
Mangevo	Lauraceae	Cryptocarya	Unknown	Tavolo	Fruit	–	0.4	0.4*	1.32
Mangevo	Lauraceae	Cryptocarya	Unknown	Tavolo	Mature leaves	–	0.4	0.4	4.79
Mangevo	Rubiaceae	Danais	sp.	Vahitamboro	Mature leaves	–	2.84	0.71	3.42
Mangevo	Moraceae	Ficus	Lutea	Amontana	Fruit	–	3.4	3.4	1.37
Mangevo	Moraceae	Ficus	reflexa	Nonoka small	Young leaves	–	0.13	0.13	5.12
Mangevo	Moraceae	Ficus	Reflexa	Nonoka small	Fruit	–	0.24	0.24	5.55
Mangevo	Clusiaceae	Garcinia	Aphanophlebia	Voamalabotaho lahy	Mature leaves	–	2.64	0.33	3.52
Mangevo	Melastomataceae	Medinilla	Unknown	Kalamasimbarika	Mature leaves	–	0.7	0.35	2.57
Mangevo	Melastomataceae	Medinilla	Unknown	Kalamasimbarika	Fruit	–	0.7	0.35*	2.59
Mangevo	Melastomataceae	Medinilla	Unknown	Kalamasimbarika	Young leaves	–	0.7	0.35	1.5
Mangevo	Asteraceae	Mikania	Unknown	Vahia	Flowers	–	0.03	0.03*	7.84
Mangevo	Rubiaceae	Mussaenda	Erectiloba	Fatora	Fruit	–	0.3	0.3*	2.11
Mangevo	Anacardiaceae	Mycronychia	Unknown	Sehana	Buds	–	0.1	0.1*	6.65
Mangevo	Lauraceae	Ocotea	Unknown	Varongy	Mature leaves	–	0.57	0.19	1.93
Mangevo	Lauraceae	Ocotea	Unknown	Varongy	Fruit	–	0.57	0.19	2.8
Mangevo	Myrsinaceae	Oncostemum	Botryoides	Kalafana large	Stem	–	0.2	0.2	2.83
Mangevo	Araliaceae	Polyscias	Unknown	Vatsilana	Young leaves	–	0.15	0.15	5.28
Mangevo	Lauraceae	Potamea	Unknown	Sary	Mature leaves	–	2.2	0.44	3.09
Mangevo	Lauraceae	Potamea	Unknown	Sary	Young leaves	–	0.2	0.2*	0.96
Mangevo	Anacadiaceae	Protorhus-Abrahamia	Unknown	Sandramy	Fruit	–	0.2	0.2*	1.91
Mangevo	Rubiaceae	Psychotria	Unknown	Fohananasity	Fruit	–	0.55	1.1*	7.05
Mangevo	-	unknown	Unknown	unknown epiphyte	Young leaves	Unknown	0.5	0.5*	2.77
Sangasanga	Moraceae	Artocarpus	Heterophyllus	Ampalibe	Fruit	Exotic	126	1.5*	6.52
Sangasanga	Burseraceae	Canarium	Madagascariensis	Ramy	Fruit	–	4.42	4.42	1.64
Sangasanga	Moraceae	Ficus	Lutea	Voara	Young leaves	–	0.49	0.49	3.95
Sangasanga	Moraceae	Ficus	Lutea	Voara	Fruit	–	0.49	0.49	1.28
Sangasanga	Moraceae	Ficus	Soroceoides (Politoria)	Nonoka large	Fruit	–	1.14	1.14	2.8
Sangasanga	Moraceae	Ficus	Trichoclada (Polyphlebia)	Nonoka small	Stick	–	0.52	0.52	6.54
Sangasanga	Moraceae	Ficus	Trichoclada (polyphlebia)	Nonoka small	Fruit	–	0.48	0.48	6.67
Sangasanga	Lauraceae	Ocotea	Cymosa	Varongy beravina	Fruit	–	3.5	3.5	0.94
Sangasanga	Strelitziaceae	Ravenala	Madagascariensis	Ravinala	Nectar	–	3.192	0.456*	2.18
Sangasanga	Arecaceae	Ravenea	Robustior	Lafa vonitra	Young leaves	–	0.4	0.4*	1.45
Sangasanga	Arecaceae	Ravenea	Robustior	Lafa vonitra	Fruit	–	6.9	6.9	4.79
Sangasanga	Euphorbiaceae	Suregada	Celastroides	Ampaliala mandindravina	Young leaves	–	0.52	0.52	7.46
Sangasanga	Moraceae	Trilepisium	Madagascariense	Ampaliala	Young leaves	–	0.52	0.52*	10.25
Sangasanga	Annonaceae	Xylopia	Buxifolia	Ramiavona	Young leaves	–	0.45	0.45*	1.03
Vatovavy	Strelitziaceae	Ravenala	Madagascariensis	Ravinala	Nectar	–	3.648	0.456*	2.42
Vatovavy	Euphorbiaceae	Uapaca	Ferruginea	Voapaka	Fruit	–	2.5	2.5*	0.99
Vatovavy	–	Unknown	Unknown	Vahy	Young leaves	Unknown	0.21	0.21	4

^a^Estimate of bite size based on other food items

Although our behavioral data are based on a relatively small number of contact hours with each group, in terms of time spent consuming different plant parts, overall they agree with long-term dietary patterns reported for each site (Additional file 1: Fig. S1). The main differences are that *Varecia* consumed less fruit and more flowers/nectar at Vatovavy during our study period compared to long-term patterns, while *Varecia* at Mangevo consumed somewhat fewer flowers during our study period compared to long-term patterns. Therefore, long-term data suggest that fruit consumption is similar across sites, and that *Varecia* at Mangevo consume more leaves and fewer flowers compared to the other two sites.

### Gut microbiome data

Although multiple samples from each individual were collected within a single season, the bacterial taxonomic composition of samples from a single individual were not more similar to each other than they were to samples from other individuals overall (unweighted UniFrac: pseudo-F_28,66_ = 0.93, p > 0.05; weighted UniFrac: pseudo-F_28,66_ = 1.1, p > 0.05). This pattern generally held within two of the three sites (Sangasanga unweighted UniFrac: pseudo-F_3,18_ = 1.2, p > 0.05; Sangasanga weighted UniFrac: pseudo-F_3,18_ = 0.75, p > 0.05; Mangevo unweighted UniFrac: pseudo-F_20,30_ = 1.2, p > 0.05), except when considering relative abundance of microbial taxa at Mangevo (Mangevo weighted UniFrac: pseudo-F_20,30_ = 1.8, r^2^ = 0.78, p = 0.02). This pattern did not hold in Vatovavy (unweighted UniFrac: pseudo-F_4,16_ = 1.8, r^2^ = 0.37, p = 0.003; weighted UniFrac: pseudo-F_4,16_ = 2.1, r^2^ = 0.41, p = 0.03).

**Fig. 5:**
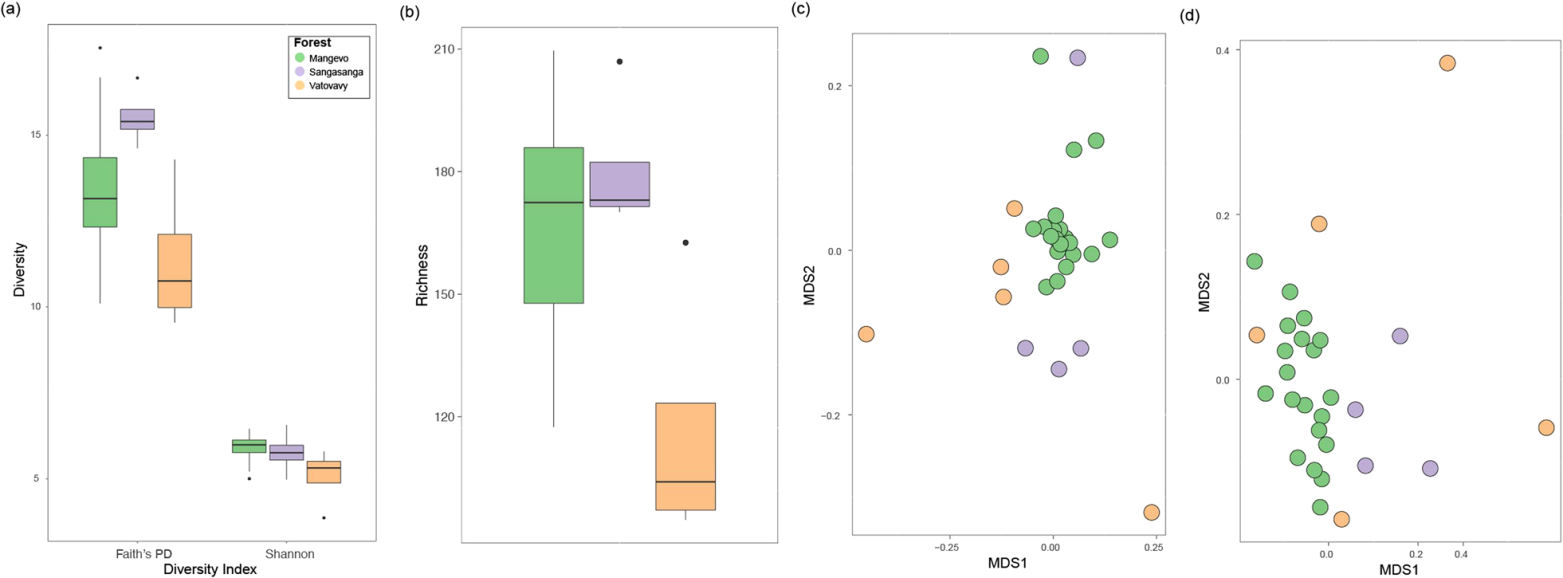
Differences in the *Varecia* gut microbiome across sites visualized using **a** boxplots of diversity and **b** richness, **c** non-metric multidimensional scaling (NMDS) of unweighted UniFrac distances, and **(d)** NMDS of weighted UniFrac distances

**Fig. 6:**
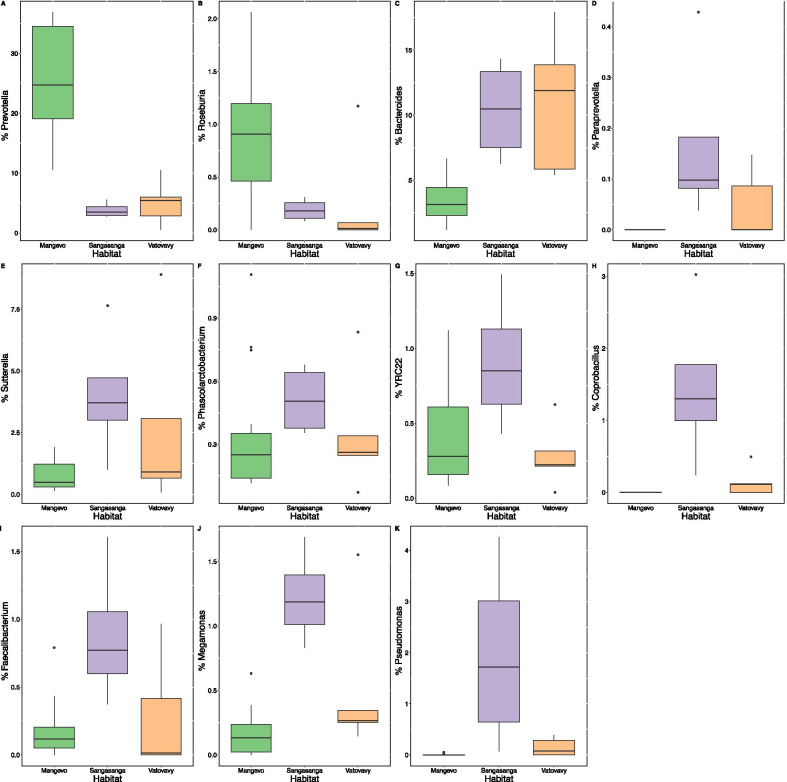
**a**–**k** Boxplots illustrating the relative abundances of microbial taxa that differed significantly across habitats

Using one randomly chosen sample per individual (n = 29 individuals), overall GM composition differed significantly across sites (unweighted UniFrac: pseudo-F_2,28_ = 2.7, r^2^ = 0.17, p < 0.001; weighted UniFrac: pseudo-F_2,28_ = 3.0, r^2^ = 0.19, p = 0.003; Fig. 5). In particular, *Varecia* at Mangevo had a distinct GM from *Varecia* at both Vatovavy and Sangasanga (Additional file 2: Table S3, Fig. 5). Microbial diversity was lowest in Vatovavy compared to both Mangevo and Sangasanga regardless of the metric utilized, and both microbial richness and phylogenetic diversity were highest in Sangasanga (richness: F_2,25_ = 7.6, p = 0.003; Faith’s PD: F_2,25_ = 5.4, p = 0.01; Shannon: F_2,25_ = 4.4, p = 0.02; Fig. 5a). The relative abundance of 56 microbial ASVs and 30 microbial genera differed significantly across sites (Additional file 2: Table S4, S5). In particular, *Varecia* in Sangasanga exhibited the highest relative abundances of *Paraprevotella, Coprobacillus*, YRC22, *Faecalibacterium, Megamonas, Phascolarctobacterium, Sutterella,* and *Pseudomonas* (Fig. 6)*. Varecia* in Vatovavy, exhibited the highest relative abundances of *Bacteroides* (Fig. 6). In contrast, *Varecia* in Mangevo exhibited the highest relative abundances of *Prevotella* and *Roseburia* and low relative abundances of *Paraprevotella, Coprobacillus,* and *Bacteroides* (Fig. 6)*.* Additionally, the absolute abundance of an unknown strain of YS2 was highest in Sangasanga and absent in Vatovavy, while an unknown strain of *Bacteroides* was highest in Vatovavy and absent in Mangevo (Additional file 2: Table S6). At the genus level, the absolute abundances of an unknown Chloroflexi, Sinobacteraceae, Sphaerochaetaceae were highest in Sangasanga, and the absolute abundance of an unknown Erysipelotrichaceae was highest in Vatovavy (Additional file 2: Table S7). Results were similar when we calculated the average GM composition for each individual from multiple samples (Additional file 1).

### Diet and gut microbiome correlation

**Fig. 7:**
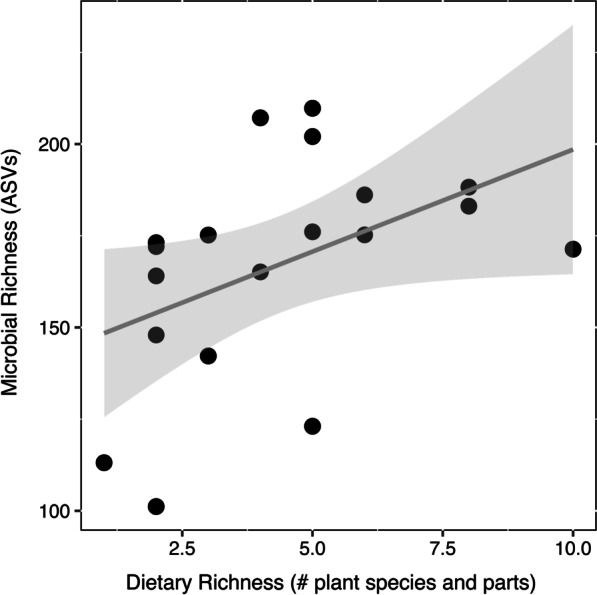
Scatterplot with a smoothed conditional mean based on linear regression demonstrating a significant positive correlation between *Varecia* dietary richness and gut microbiome richness

**Fig. 8:**
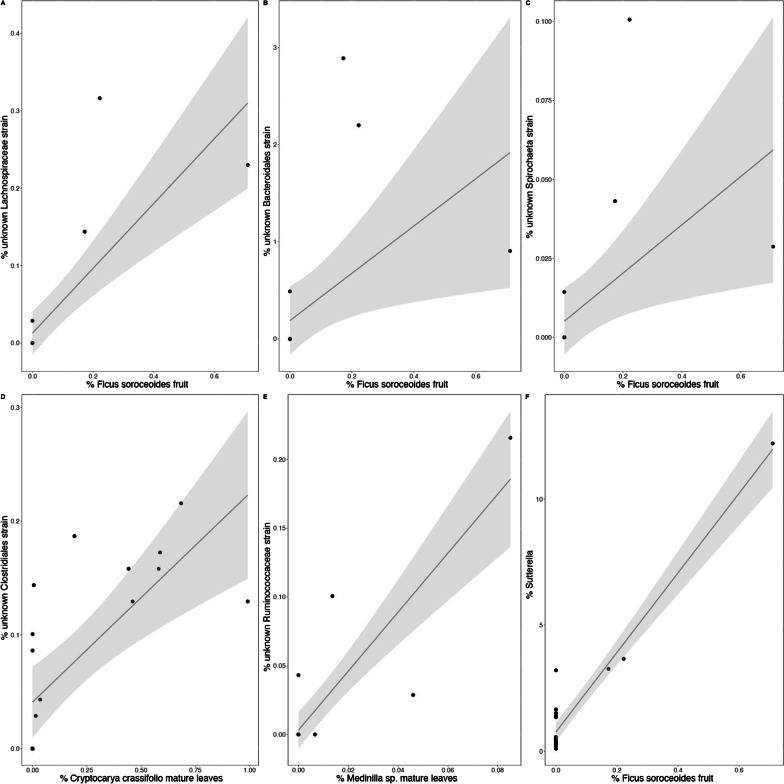
**a**–**f** Scatterplots with smoothed conditional means based on linear regression showing positive correlations between *Varecia* intake of specific food items and the relative abundances of individual microbial taxa

Using GM data from only the samples for which we collected host behavioral data simultaneously, Mantel tests showed a positive correlation between the overall *Varecia* diet and overall gut microbiota composition (Mantel r = 0.56, p < 0.001). Overall dietary richness was a significant predictor of GM richness (F_1,17_ = 4.4, p = 0.05; Fig. 7). After FDR correction, *Ficus soroceoides* fruit consumption was positively correlated with the relative abundances of an unknown Lachnospiraceae strain (r = 0.90), an unknown Bacteroidales strain (r = 0.85), an unknown *Sphaerochaeta* strain (r = 0.75), and the genus *Sutterella* (r = 0.82; Fig. 8). *Cryptocarya crassifolia* mature leaf consumption was positively correlated with the relative abundance of an unknown *Clostridiales* strain (r = 0.82), and *Medinilla* sp. mature leaf consumption was positively correlated with the relative abundance of an unknown Ruminococcaceae strain (r = 0.93; Fig. 8). All of these microbial strains except the unknown Ruminocacceae and *Spirochaeta* also exhibited significantly different relative abundances across sites.

## Discussion

To build upon growing interest in using the GM as a conservation tool, here we used an endangered lemur, *Varecia variegata*, to explore the relationship between habitat disturbance, diet, and GM composition. As hypothesized, we found significant differences in *Varecia* dietary composition (i.e., plant parts consumed), richness (i.e., the number of taxa consumed), and GM composition and richness across habitats with distinct anthropogenic disturbance exposures. Patterns in *Varecia* diet and microbiome composition were correlated. However, in contrast to expectations, *Varecia* occupying the site qualitatively categorized as ‘heavily disturbed’ had higher dietary and microbial richness than *Varecia* occupying the ‘moderately disturbed’ site. Furthermore, diet did not explain all of the variation in *Varecia* microbiome composition across populations. Overall, these findings emphasize the importance of quantitatively characterizing diet and other environmental factors when examining the influence of anthropogenic habitat disturbance on the GM. They also confirm that diet-GM interactions warrant further consideration when developing applied conservation efforts.

While many existing microbiome studies that sample wild animals across habitats do not include detailed behavioral data, our findings suggest these data are important for more accurately understanding host-microbe interactions in the context of environmental change. First, behavioral data allowed us to identify complex patterns in *Varecia* diet across habitats that we otherwise would have overlooked. For example, while *Varecia* at Mangevo accessed more unique food items as a population, in accordance with the expectation that undisturbed habitats host the highest diversity of potential food items, at the individual level, both dietary richness and fruit consumption were highest in Sangasanga, the most disturbed site. It is possible that these differences observed at the individual level reflect microhabitat differences in range use. Members of the anthropogenically disturbed Sangasanga and Vatovavy populations may interact with a less floristically diverse, more homogeneous habitat than do the Mangevo *Varecia*, resulting in less inter-individual variation in diet. In contrast, Mangevo boasts more floristic diversity, as well as a greater heterogeneity in microhabitats [53](Baden & Mancini, unpublished data). Recent work has found that members of the Mangevo population utilize small, only minimally overlapping home ranges with other members throughout their community [54], and that individual home ranges vary in floristic diversity and quality (Baden, unpublished data), lending support to this hypothesis.

Similarly, while we expected reduced fruit consumption and increased leaf consumption in more disturbed habitats, our data did not support this hypothesis, even when we considered dietary data collected across several years. Because dietary overlap was low across habitats, these patterns may still underlie differential effects habitat disturbance has on plant community assemblages. For instance, animals from both Vatovavy and Sangasanga habitats relied heavily on the flowers and nectar of *Ravenala madagascariensis*, the native traveler’s palm, whereas the animals from Mangevo were never documented consuming these food items despite having access to them. The different forms (i.e., subspecies) of this palm vary across habitat types: the *bemavo* form found in Vatovavy and Sangasanga is characteristic of open forests and anthropogenically modified landscapes while the *malama* form identified in Ranomafana National Park is adapted to the microclimate of the forest floor in the understory of undisturbed rainforests [55]. Differences in flower and nectar nutritional quality are poorly understood, but it is possible that *Varecia* preferentially use the *bemavo* form as a result of some improved nutritional trait. It is also possible that *Varecia* only consume the palm, in any form, in the absence of other key food items to fill a nutritional gap. The availability and nutritional content of other plant species and plant parts are also likely to vary across habitats, making it difficult to predict dietary diversity and/or fruit content based on qualitative assessments of habitat disturbance.

Detailed dietary data also provided important context for our microbial data. As expected, *Varecia* diet composition and richness were correlated with microbiome composition and richness, suggesting that diet plays an important role in shaping *Varecia* microbiomes across habitats. These findings mirror a previous study of the gut microbiome of black howler monkeys (*Alouatta pigra*) across habitats in Mexico that included quantitative dietary data [*A. pigra*, 16]. However, in the present study, because our assumption that dietary richness would be lowest in the most qualitatively disturbed habitat was not supported, neither was our assumption that microbial richness would be lowest. Without quantitative dietary data, the patterns observed in the microbial data would have been more difficult to interpret.

Overall, the relationships that we identified between *Varecia* diet and gut microbiome composition provide an important foundation for understanding the response of the *Varecia* microbiome to habitat change. Beyond demonstrating the importance of dietary diversity in maintaining microbiome diversity, our data allowed us to identify three food items that appear to most strongly influence the *Varecia* gut microbiome: *Ficus soroceoides* fruit, *Cryptocarya crassifolia* mature leaf, and *Medinilla* sp. mature leaf. These relationships may be a result of *Varecia* reliance on microbial pathways to digest these foods more efficiently. Leaves consumed by *Varecia* tend to have higher fiber content compared to other food items, and *F. soroceoides* fruits have higher fiber content compared to many other fruits [56]. Microbes belonging to the orders Bacteroidales and Clostridiales, which includes the families Ruminococcaceae and Lachnospiraceae, are known fiber degraders [57, 58], and their relative abundances varied most in response to the intake of these food items. Functional links between other microbial taxa that exhibited changes in relative abundance associated with the intake of these foods, such as *Sphaerochaeta* and *Sutterella,* are less clear. More data describing *Varecia* food nutritional content as well as the functions of these microbial strains will be critical to improving insight into these fine-scale host-microbe interactions (Beeby & Baden, unpublished data). Our understanding of the functions of known microbial taxa remains limited across the animal kingdom [59], and more than 60% of the microbial sequences identified in lemur fecal samples are unknown at the genus level, and as many as 40% are unknown at the phylum level [29]. Also, because three of these foods were only available at one site during the study period, the observed correlations between their consumption and GM composition were driven by variation in the quantity consumed among individuals within a given site. Subsequent studies that include more individuals across a longer period of time will be necessary to determine the extent to which these correlations are maintained both across seasons and across sites. However, our findings suggest that these food items could eventually be useful for conservation efforts targeting the *Varecia* GM. If the microbial taxa they are associated with have beneficial effects on hosts, prioritizing the inclusion of these plant species into habitats could improve *Varecia* health. Alternatively, excluding these plants could also improve *Varecia* health if the associated microbial taxa have detrimental effects on hosts.

While diet had a strong effect on multiple measures of *Varecia* microbiome composition, our data suggest that other factors are also likely to contribute to differences in the *Varecia* microbiome across habitats. Diet did not explain all of the variation in our dataset, and we detected differences in the relative abundances of microbial taxa across habitats that were not directly correlated with diet. Some of these differences may be a result of dietary variables that we were unable to measure during the relatively short study period. For example, *Roseburia* and *Prevotella* are important fiber degraders [58] and had the highest relative abundances in Mangevo, where fiber-rich leaf consumption was also highest. However, other patterns may be a result of other environmental factors that vary across sites and in response to different types of habitat disturbance, such as exposure to humans and livestock or increased population densities and associated social stress. For example, because Sangasanga is used to grow shade coffee, it is also more heavily managed, and *Varecia* are exposed to more frequent human contact. Interestingly, *Faecalibacterium* and *Sutterella* have been identified as microbial taxa that are characteristic of humans [60, 61], and these taxa were most abundant in Sangasanga. YRCC relative abundances were also elevated in Sangasanga, and this taxon is common in livestock [62–64]. Sampling across more sites will be necessary to disentangle the effects of these distinct environmental factors on the GM. Nevertheless, our data provide evidence that diet is unlikely to be the only factor affecting the GMs of wild non-human primates exposed to various forms of anthropogenic habitat disturbance.

It is difficult to make strong predictions about the health impacts of habitat degradation that could be mediated by the *Varecia* GM given the previously-noted limitations in functional knowledge of lemur gut microbial taxa as well as the fact that we could not collect data describing *Varecia* physiology and health for this study. Nevertheless, some of the observed patterns suggest that there may be health consequences of these microbial differences. For example, *Sutterella* and *Psuedomonas* were most abundant in Sangasanga and have been associated with disease in some contexts in humans [65]. Additionally, increased *Bacteroides* relative abundances, which were observed in both degraded habitats, have been reported in captive non-human primates, have been associated with diets high in fat and protein in humans, and are sometimes used as a marker for increased metabolic disease risk [66, 67]. In contrast, *Prevotella* and *Roseburia* are generally considered to be indicators of a ‘healthy’ GM with reduced disease risk [66, 67], and these taxa were most abundant in Mangevo, our least disturbed site. Moving forward, additional data will help distinguish which GM shifts represent local adaptations to habitat characteristics and which signal health risks.

Although previous studies have reported some influences of habitat disturbance on mammalian GMs [16, 30, 31, 37, 68], direct comparisons with our findings are unlikely to provide robust insight based on the current state of the literature. Most existing studies of the effects of habitat disturbance on the GM do not quantitatively describe disturbance despite the fact that disturbance is likely to manifest itself differently at different sites and for different host species. Therefore, it is extremely challenging to determine the extent to which similar environment–GM interactions are being compared across studies. Although there are likely to be generalizable patterns through which different processes of disturbance alter the GM, we cannot begin to identify them without paired quantitative environmental and GM data from multiple sites and species.

## Conclusions

Overall, our results show that anthropogenic habitat disturbance affects the GM of *Varecia,* a Critically Endangered, fruit-specialist lemur, but that broad categorical descriptions of disturbance are not useful predictors of *Varecia* GM composition. Additionally, while diet appears to be a major contributor to the observed GM patterns, it cannot fully explain them. These findings are likely to be generalizable across a variety of primate species and point to key gaps in conservation-based GM research more broadly. Qualitative descriptions of habitats limit the applied utility of many existing studies. GM surveys must be combined with detailed data describing the local manifestations of disturbance as well as host physiological status. Once generated, this information can be used to develop microbial biomarkers of environmental change for a range of animal populations and, ultimately, provide novel targets for both habitat restoration and health interventions. The current study provides an important foundation for this approach in *Varecia* and will hopefully serve as a model for developing similar studies in more wild mammal species globally.

## Methods

### Forest site descriptions

We sampled *Varecia* at three sites with similar climates but different amounts and types of human impact (Table 1). Mangevo is a primary rainforest site with little evidence of human impact; signs of livestock, logging, and/or fire are rare [69]. It lies within the southern parcel of Ranomafana National Park, which protects 41,600 ha of montane rainforest within the larger Ambositra-Vondrozo Corridor (COFAV) [70, 71]. Vatovavy is a moderately disturbed 353 ha forest fragment located approximately 72 km southeast of Mangevo. It has been subject to logging, although a dense underbrush signals regrowth [72]. There is increased canopy openness compared to Mangevo as well as an altered plant community structure (Table 1). Sangasanga, the heavily disturbed site, is located within 6 km of Vatovavy and is a 99 ha forest fragment, a portion of which is used as a coffee plantation where regrowth is cut back regularly [72]. While canopy openness and plant community structure are similar to that of Vatovavy, Sangasanga is much closer to human settlements as well as other forest fragments (Table 1).

### Behavioral data collection

Data collection occurred from June 2018 through August 2018 at Mangevo (21° 22′ 59″ S, 47° 28′ 0″ E), Vatovavy (21° 24′ 20″ S, 47° 56′ 27″ E), and Sangasanga (21° 21′ 43″ S, 47° 50′ 54″ E). During this period, we collected behavioral data at each site using full-day continuous focal follows of radio-collared individuals. Observations were conducted at each site consecutively for three weeks, beginning with Mangevo and ending with Sangasanga. Due to logistical constraints, we followed individuals at Mangevo from 7:30 to 16:30 (N = 12 individuals, n = 99.3 observation hours; Additional file 2: Table S8) while in Vatovavy and Sangasanga, we followed individuals from 8:30 to 14:30 (N = 4 individuals at each site, 27.0 and 31.7 observation hours, respectively; Additional file 2: Table S8). To account for this bias, we only considered data from approximately 8:15 to 15:00 at each site (n = 136.9 observation hours) for subsequent analysis (Additional file 2: Table S8). Individuals were observed once each at Mangevo and multiple times at Vatovavy and Sangasanga, but to make the data comparable, we chose to include data from one focal follow randomly for each individual at the latter two sites. We recorded time spent resting, traveling, feeding, feeding out of sight, engaging in social interactions, and out of sight.

### Feeding data collection

During feeding bouts we recorded the plant species, plant part, and stage of ripeness (ripe or unripe). We described dietary richness for each group by summing the total number of unique food items (plant part and plant species) *Varecia* was observed consuming at each site, as well as calculating the average number of food items consumed by individuals in the group. We described diet composition in terms of proportion of grams consumed of each food item. To do this, we recorded consumption rate for each food item (number of bites taken per bout). At the end of the study period in each forest, we collected, measured, and weighed thirty samples of each plant part consumed by *Varecia*, often from the same plants that had been utilized during focal sampling. We divided the average weight of each food item by the number of bites required to consume it. We then multiplied the number of minutes spent consuming the food item by the bite rate in grams. Because nectar was an important part of the *Varecia* diet during this period, we measured the milliliters of nectar in each flower (*Ravenala madagascariensis*). The nectar has a fourteen percent sucrose concentration, or fourteen grams of sugar per one-hundred milliliters of nectar [73]. We used this value to estimate the average grams of sugar per flower and combined it with the average grams of water per flower (converted from milliliters to grams with a one-to-one ratio), and multiplied this by the number of flowers consumed. Finally, for ‘feeding out of sight’, we used bite rates from a similar plant species. The plant species consumed as well as their estimated grams per bite are reported in Table 2.

### Fecal sample collection

We collected fecal samples from 20 individuals in Mangevo (N = 30 samples), five individuals in Vatovavy (N = 17 samples), and four individuals in Sangasanga (N = 20 samples; Additional file 2: Table S8). These included samples from our focal individuals at each site, as well as samples collected from other individuals opportunistically. Samples were collected immediately following defecation and stored in 99% ethanol at room temperature until transport to the Amato lab (~ 1 month), where they were stored at − 80 °C until processing.

### Gastrointestinal microbiome data extraction

We extracted DNA from fecal samples (Qiagen PowerSoil DNA Isolation Kit) and amplified the V4-V5 region of the 16S rRNA gene with the 515F/926R primers, using previously described PCR protocols [74, 75]. One sample that could not be amplified was discarded, and both extraction and PCR negatives were used for quality control. PCR products were purified, normalized, and sequenced (Illumina MiSeq with V4 chemistry) at the DNA Services Center at the University of Illinois at Chicago. To be able to transform 16S relative abundance data into count data, qPCR of the 16S rRNA gene was performed on our samples by the DNA Services Center as previously described [76, 77].

Sequencing yielded 1,630,313 raw sequence reads (average 24,333 sequences/sample, range: 18,820 to 36,634 sequences/sample). We quality filtered raw sequence data and identified amplicon sequence variants (ASVs) using the default settings of the DADA2 plug-in [78] for QIIME2 (v2019.7) [79]. Taxonomy was assigned in QIIME2 using a Naive Bayes classifier trained on the Greengenes 13_8 99% OTU database using the full 16S rRNA gene sequence lengths. Mitochondria and chloroplast ASVs were filtered from the dataset. After quality filtering, there was an average of 11,941 sequences/sample (range 6956 to 29,294).

Alpha rarefaction indicated that all samples had sufficient sequencing coverage. Therefore, we used the breakaway plug-in in QIIME2 to estimate the taxonomic richness of all samples and the diversity plug-in to calculate Shannon and Faith’s Phylogenetic diversity. Breakaway indicated that four samples had an error greater than ten (VVAR.MADA.18.NM.18, VVAR.MADA.18.NM.37, VVAR.MADA.18.NM.60, VVAR.MADA.18.NM.61), so we removed these samples from diversity statistical analyses. We used the core-metrics-phylogenetic plug-in in QIIME2 to rarefy the data to 6956 reads/sample and generate unweighted and weighted UniFrac distance matrices. Finally, we used our 16S qPCR data to calculate the absolute abundances of all microbial taxa in our samples as previously described [77].

### Statistical analysis

We evaluated differences in proportion of time spent in each activity and dietary richness across sites using an ANOVA. Variation in percent of total grams of each food item consumed was assessed using a Kruskal–Wallis rank-sum test due to non-normal data distributions.

To test differences in overall GM taxonomic composition across sites, we used the adonis2 package in vegan [80] to run a permutational analysis of variance (PERMANOVA) on unweighted UniFrac and weighted UniFrac distance matrices. We tested pairwise site differences with PairwiseAdonis [81]. We evaluated differences in microbial richness and diversity across sites using ANOVA. We used a series of linear regressions to test for differences in both the relative and absolute abundance of individual GM taxa across sites at the ASV and genus level. All p values were corrected for multiple tests (fdrtool, R v. 3.5.4). Because we had multiple samples per individual, we tested for the effect of individual on GM composition before proceeding with other analyses. Based on these results, we performed all analyses using a randomly selected sample from each individual (Additional file 2: Table S9). However, we also repeated analyses using average GM composition values for each individual to ensure we were not introducing bias.

With the subset of samples for which we had paired diet and GM data (Additional file 2: Table S9), we used a Mantel test to explore the correlation between overall *Varecia* diet composition and GM composition. We performed linear regressions to test for an association between dietary richness and GM richness. Finally, we used CCREPE [82] to test for correlations between the dietary percentage of nine food items consumed by more than two individuals and the relative abundance of all microbial ASVs. This package is designed for compositional datasets like ours and incorporates an FDR correction for multiple tests.

## Supplementary Information

**Additional file 1: Figure S1.** Average percent time (±SD) individuals spent consuming each major plant part in the *Varecia *diet at each site (a) based on long-term data collected between 2010 and 2019 across all months (n=4,228 observation hours at Mangevo; n=2,753 hours at Vatovavy;and n=3,483 hours at Sangasanga) and (b) during the study period. Long-termdietary data provided by ALB (Mangevo) and SMH, EEL, SEJ (Sangasangana,Vatovavy).

**Additional file 2: Table S1.** ANOVA statistics for differences in activity budget (% time) across sites. **Table S2.** Kruskal-Wallis statistics for differences in diet composition (% grams) across sites. **Table S3.** PairwiseAdonis statistics for differences in microbiome composition across sites using both unweighted and weighted UniFrac distances. **Table S4.** Average relative abundance (+/- SD) of microbial ASVs that differed in relative abundance across sites. **Table S5.** Average relative abundance (+/- SD) of microbial of microbial genera that differed in relative abundance across sites. **Table S6.** Average absolute abundance (+/- SD) of microbial ASVs that differed in absolute abundance across sites. **Table S7.** Average absolute abundance (+/- SD) of microbial genera that differed in absolute abundance across sites. **Table S8.** Summary of data collected per individual at each site. **Table S9.** Full list of samples analyzed.

## Data Availability

Raw sequence data can be obtained from the NCBI Sequence Read Archive (SRA) under BioProject PRJNA775472. Ecological data will be provided upon request. No custom code was used, and all analyses are described in the main text. The authors can provide additional information upon request.

## Abbreviations

GM: Gut microbiome
PCR: Polymerase chain reaction
qPCR: Quantitative polymerase chain reaction
ASV: Amplicon sequence variant
ANOVA: Analysis of variance
PERMANOVA: Permutational analysis of variance
NMDS: Non-metric multi-dimensional scaling
