# Supplementary material for: The gut microbiome as an indicator of habitat disturbance in a Critically Endangered lemur
Source: BMC Ecol Evol. 2021 Dec 16;21:222. doi: 10.1186/s12862-021-01945-z (PMC8680155; doi:10.1186/s12862-021-01945-z)
Supplement: Supplementary file 1 — Additional file 1: Figure S1. Average percent time (±SD) individuals spent consuming each major plant part in the Varecia diet at each site (a) based on long-term data collected between 2010 and 2019 across all months (n=4,228 observation hours at Mangevo; n=2,753 hours at Vatovavy;and n=3,483 hours at Sangasanga) and (b) during the study period. Long-termdietary data provided by ALB (Mangevo) and SMH, EEL, SEJ (Sangasangana,Vatovavy). [file 12862_2021_1945_MOESM1_ESM.docx]

**Supplemental Material**

*Results using average values per individual* Using average values for microbial taxa prevalence and relative abundance for individuals that were sampled multiple times, we detected differences in overall gut microbiome composition (unweighted UniFrac: F_2,27_ = 4.4, r^2^ = 0.26, p < 0.001, weighted UniFrac: F_2,27_ = 8.2, r^2^ = 0.40, p < 0.001). Microbial richness and diversity also differed across sites regardless of the metric used (ASV richness: F_2,25_ = 8.3, p = 0.002; Faith’s PD: F_2,25_ = 7.6, p = 0.003; Shannon: F_2,25_ = 13.9, p < 0.001). The relative abundances of 1747 ASVs were different across habitats (Table S8), and the relative abundances of 335 genera were different across habitats (Table S9). Dietary composition and microbiome composition were strongly correlated (Mantel r = 0.30, p = 0.004), as were dietary richness and microbiome richness (t=5.2, df = 17, r = 0.78, p < 0.001).

Figure S1. Average percent time (±SD) individuals spent consuming each major plant part in the *Varecia* diet at each site (a) based on long-term data collected between 2010 and 2019 across all months (n=4,228 observation hours at Mangevo; n=2,753 hours at Vatovavy; and n=3,483 hours at Sangasanga) and (b) during the study period. Long-term dietary data provided by ALB (Mangevo) and SMH, EEL, SEJ (Sangasangana, Vatovavy).

**
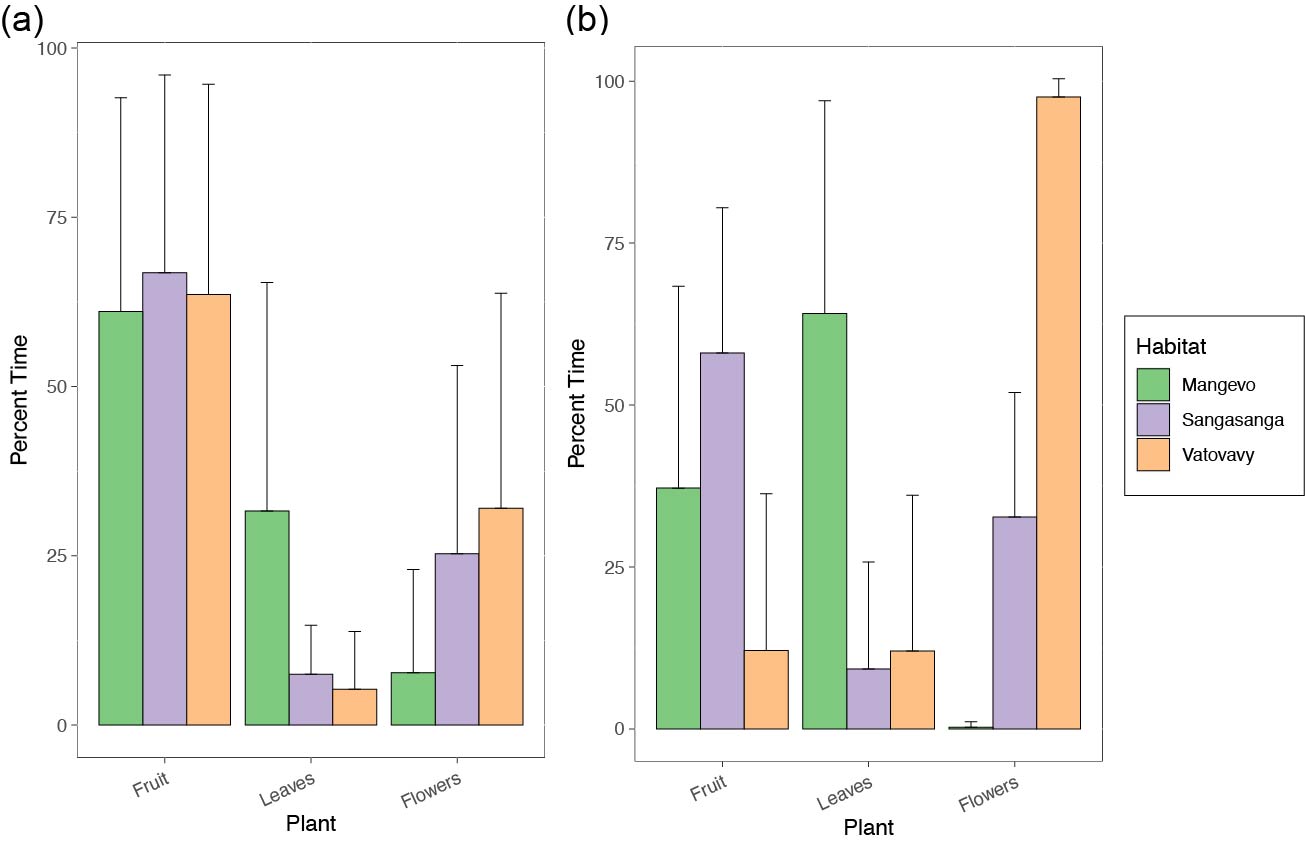
**
